# Cases of Vagismus, with the Method of Treatment

**Published:** 1862-06

**Authors:** J. Marion Sims


### CASES OF VAGINISMUS, WITH THE METHOD OF TREATMENT.

By J. MARION SIMS, M.D.

Reprinted from the Bulletin of the New York Academy of Medicine.

In May, 1857, I was called to see a lady, 45 years of age, who, married at 20, had been an invalid ever since. Menstruation, always painful, had just ceased. She had great irritability of the bladder, a sense of bearing down, and other symptoms of uterine derangement. But the most remarkable thing in her history was the fact that she had remained a virgin, notwithstanding a married state of a quarter of a century. Some two or three years after marriage, her physician discovered a sanguineous tubercle at the meatus urinarius, and removed it with the expectation of relieving her peculiar condition, but no benefit ensued. He then attempted to dilate the vagina with graduated bougies, which produced the most intolerable suffering without the slightest permanent improvement. She next consulted the most eminent physicians in the principal capitals of America, and visited London, Paris, and other European centres of learning, asking advice of leading surgeons, but no one could give a satisfactory solution of the case, or advised anything more than the bougie system, which had been already fruitlessly exhausted. Possessed of ample means, she and her husband had left nothing untried that promised the least hope of success. And thus many, many long years had passed when

I was sent for, not to be consulted in respect to this peculiarity, which they had long since learned to look upon as incurable, but for the state of her general health.

I found her nervous system in a deplorable condition. It was exceedingly impressible, the slightest noise causing her intense pain. She was only able to walk across the room, but did not often venture even upon this, being confined for the most of the time to her couch, where she gave herself up to unceasing intellectual effort. Her mental tension and sedentary habits were supposed to be the cause of her great nervousness.

Amongst other means of diagnosis, I proposed a vaginal examination, which she assured me was impossible, then gave me the history already related. I attempted it, however, but failed completely. The slightest touch at the mouth of the vagina produced the most intense agony, throwing her nervous system into great agitation, with general muscular spasm and shivering of the whole frame, as if with the rigors of an intermittent, while she shrieked aloud, her eyes glaring wildly, and tears rolled down her cheeks, all rendering her a pitiable object of terror and suffering. Notwithstanding all these outward involuntary evidences of physical commotion, she had moral fortitude enough to hold herself on the couch, imploring me meanwhile not to desist from my efforts while the least hope remained of finding out anything about her inexplicable condition. After pressing with all my strength for some minutes, I succeeded in introducing the index finger into the vagina, up to the second joint, but no further. The resistence to the passage was so great, and the vaginal contraction so firm, as to deaden the sensation of the finger, and thus the examination revealed only an insuperable spasm of the sphincter vaginæ. Whether the vagina was defectively developed or normal, I could not determine. I candidly told her husband that I knew nothing whatever about the case, that I had never seen or heard of anything like it, and that it would be quite presumptuous in me to hazard an opinion, or hope to do anything for her, when they had consulted the ablest surgeons in the world without receiving the least information on the subject, and that I could promise nothing. However, I suggested the propriety of her going to New York for further investigation under anæsthesia. She accordingly did so, and I invited the late Dr. John W. Francis, Dr. Emmet, of the Woman's Hospital, Prof. Van Buren, and Dr. R. S. Kissam, to see her. The two last named gentlemen assumed the responsibility of the etherization, which was to me a matter of some anxiety, owing to her peculiar nervous organism. Previously to the anæsthesia, I attempted to make a vaginal examination, when the same train of symptoms was manifested as on the former occasion. But as soon as she was fully under the influence of the ether, greatly to my surprise, I found the mouth of the vagina completely relaxed, and the vagina itself perfectly normal, not presenting the least deviation from health. It was not large, but certainly quite as well developed as it ought to be at her time of life, and under the circumstances. The uterus was retroverted, and there was a small polypoid excresence about as large as a pea hanging from the os tincæ. This was removed, not with the expectation that it would have any influence upon her condition, but to prevent the risk of future growth.

The opinion that I gave on the case was this: that it was a spasmodic contraction of the sphincter vaginæ, resulting from an irritable condition of the nerves of the part, which I could not explain. To the question whether it were possible to effect a cure, I replied that I did not know, for the books threw no light on the subject; but that the only rational treatment appeared to me to be surgical, *i. e.* dividing the muscles and nerves of the vulval opening. They seized on the idea, and insisted on the operation, which I declined to perform, on the ground that an untried process was not justifiable on one in her position in social life, the hospital being the legitimate field for experimental observation.

I have related the case somewhat at length, to make it descriptive of the class which it represents, and I shall be glad if this learned body will allow me, in my own simple way, to continue the story of my own experience in the matter. I have nothing to say on the literature of the subject; I leave that to others.

The high intellectual endowments of this lady, her elegant culture and fine social position, as well as her long suffering, all conspired to make her case one of much thought and anxiety to me, and I could not easily dismiss it from my mind. I consulted authors, and found cases described by them of pruritis, hyperæsthesia, neuralgia, neurosis, artresia, etc. etc., all of which I had seen, but nowhere did I find any description of disease answering to the peculiarities of this case, which I naturally concluded to be unique and anomalous. But about fifteen months afterwards, Professor Pitcher, of Detroit, Michigan, sent me another case, precisely similar, except that the lady had been married for two years. She had the same instinctive dread of being touched, the same muscular contraction of the whole frame, etc., while it was utterly impossible to pass the finger into the vagina. As the lady's husband threatened to obtain a divorce, I looked upon her case as justifying the experiment. So, fully explaining to her our ignorance on the subject, I proposed a series of experimental incisions, etc. etc., to which she readily consented. Thinking the division of the irritable spasmodic outlet to be the only rational operative procedure, I at first divided only the edges of the hymeneal membrane on each side of the fourchette. No relief ensued. After waiting for the wounds to heal, I divided the parts again at the same points, extending the incisions deeply, however, through the mucous membrane, and through some of the fibres of the sphincter muscle. This was followed by some improvement; she could bear the introduction of one finger without great pain, and could even tolerate two, but with considerable suffering. I now saw that the hymen itself was the focus of the excessive sensibility, and proposed to cut it out entirely, and afterwards to repeat the lateral incisions as before, making them deeper, and rendering the dilation permanent by the use of a properly constructed vaginal dilator. By this time the mother of the lady had come to the very just conclusion that I was *experimenting* on her daughter. I told her that it was true, and attempted to explain to her the propriety of such a course when a lawsuit and divorce were in prospective. The mother, however, was inexorable, and unfortunately removed her daughter from my care. Nevertheless, her improvement was so great that I have no doubt of her fulfilling the relation of wife under some difficulties.

The experience gained by this case was of great value to me. A few weeks afterwards, singularly enough, another case fell into my hands—the wife of a clergyman, who had been married for six years. Sexual intercourse was impossible. Several surgeons had been consulted, without receiving any explanation of the case, and, of course, without relief. On examination, I discovered a sanguineous, mucous, irritable tumor at the mouth of the meatus urinarius, and notwithstanding the experiments already related, persuaded myself that this tubercle was the cause of all the trouble. The tumor was removed and its seat cauterized. In due time, she returned home, but came back in a few days, to report a persistent state of virginity. On a more minute examination, I found the case to be, in all particulars, precisely like those previously related, but not quite so intense in its manifestations. The slightest touch at the reduplication of the hymeneal membrane with a feather or a camel's hair pencil, produced as severe suffering as if she were cut with a knife.

While this lady was under treatment (April, 1859), a fourth case came under my observation. The lady had been married three years. Sexual intercourse had been imperfectly accomplished a few times during the first few weeks after marriage. She innocently supposed that all women had to suffer as she did, and tried to bear it like a good Christian, but her sufferings were so intense that she at last looked with the greatest terror on the approaches of her husband, to whom she was devotedly attached. At her earnest entreaties, her husband, who was equally devoted to his wife, ceased all efforts at sexual intercourse, and they lived and loved as innocently as two little children. But at length the mother of the poor timid girl began to wonder why, after three years of marriage, her daughter, who seemed to be healthy, and who had a healthy, vigorous, young husband, had not become pregnant, and ventured to speak of her disappointment in not being advanced to the honorable title of grandmother. Upon this, the daughter hesitatingly explained the whole to the mother, who immediately brought her to me. I found precisely the same condition of things as already described.

Three weeks after this, my friend, Dr. Harris, of E. 30th st., New York, brought me another (the fifth) case. The patient had been married two and a half years, and, in consequence of her persistent virginity, her husband was truly unhappy. I had now (June, 1859) three cases under observation at the same time. To cut short this long narrative, I will simply say that after many experiments and disappointments, all were perfectly cured in August, 1859.

From personal observation, I confidently assert that I know of no disease capable of producing so much unhappiness to both parties to the marriage contract, and I am happy to state that I know of no serious trouble that can be so easily, so safely, and so certainly cured. I now venture, with the approbation of this learned body, to give this affection a name as well as a remedy.

By the term Blepharismus or Blepharospasmus, we mean an involuntary, painful, spasmodic contraction of the orbicularis palpebrarum, with great supersensitiveness or intolerance of light. By the term Laryngismus, we mean a spasmodic contraction of the rima glottidis, with stridulous inspiration. And by the term Vaginismus, I propose to designate an involuntary, spasmodic closure of the mouth of the vagina, attended with such excessive supersensitiveness as to form a complete barrier to coition. These various affections may or may not be complicated with inflammation, but do not necessarily depend upon it. We may have vesical tenesmus without inflammation of the bladder, and rectal tenesmus without rectitis. The most perfect examples of Vaginismus that I have ever seen have been uncomplicated with inflammation; but I have met with cases in which a slight redness or erythema was visible at the fourchette, just without the reduplication of the vaginal mucous membrane, called the hymen. Usually, the hymen is thick and voluminous, and, when the finger is passed into the vagina, its free border often feels as resistant as if bound by a fine cord or wire; but it may also be firm and unyielding, with even the wire-feeling free border, without symtoms of Vaginismus. There need be no mistake in diagnosis. It can be confounded only with impermeable hymen or with atresia. In each of these, marriage may have existed without consummation, but the true cause becomes patent on investigation.

In a case of Vaginismus, the gentlest touch with the finger, a probe, or ever a feather, produces the most excruciating agony. The sensitiveness is at all parts of the vaginal outlet, is very great at the meatus urinarius, and on each side of it, just where the hymen takes its orign; is greater still on the vulval or outer face of the hymen, near the orifice of the vulvo-vaginal gland, and greatest at the sulcus or reduplication from the vulval orifice. Often, the most sensitive point of all is at the fourchette, where the hymen projects upwards. I have often heard patients shriek with terror and agony, exclaiming that I was thrusting a dagger into the body, when I merely touched the sensitive points with a camel's hair pencil or a soft feather; and, again, these same patients have declared that they felt comparatively nothing when I have had the parts held asunder, so as to pass a probe into the vagina, making a forcible pressure against the internal or vaginal surface of the hymen, thus proving that, while the outer face of the hymen was supersensitive, its inner surface was normal. In all cases, the mere spasm of the sphincter is painful, and in many cases the sphincter ani feels almost as hard as a ball of ivory. Indeed, one of my patients supposed it to be a tumor to be cut out before she could be cured. The spasm of the sphincter is pathognomonic of the disease; the supersensitiveness, diagnostic. The fact is more delicately shown by touching the outer surface of the hymen, particularly at its reduplication, with a soft camel's hair pencil.

*Treatment.*—I shall not detain you with a rehearsal of the steps by which the proper treatment was finally determined; enough has been said already to show that it was not accidental, as my observations extended from May, 1857, to August, 1859. The treatment consists in the removal of the hymen, the incision of the vaginal orifice, and subsequent dilatation. The last is utterly useless without the others, but is essential to easy and perfect success with them.

I usually make two operations, though all may be done in one. Placing the patient, etherized, on the left side, I seize the hymeneal membrane with a pair of forceps, just at its junction with the urethra on the left side, and, putting it on the stretch, clip it with properly curved scissors till the whole of it is removed in one continuous piece. In some cases the hæmorrhage is sufficient to require a compress of lint, thrust into the mouth of the vagina, while in others it is unimportant. In two instances the bleeding was excessive, but was easily controlled by the liquor ferri persulphatis. The cut usually heals in three or four days, after which the operation for radical cure may be performed.

Notwithstanding the removal of the thick, sensitive hymen, the citatrix marking the original place at the mouth of the vagina, is excessively sensitive, and, in some instances, feels hard and tense, as if a small cord were constricting the outlet. This I formerly divided at different points, and in divers ways, during the course of my experiments, and finally arrived at the following method as being the surest and best:—

Place the patient, fully etherized, on the back, as in the position for lithotomy, pass the index and middle fingers of the left hand into the vagina, separate them laterally so as to open the vagina as widely as possible, putting the fourchette well on the stretch. Then make a deep cut with a common scalpel through the vaginal tissue on the right of the mesial line, bringing it from above downwards, and terminating at the raphe of the perineum. This cut forms one side, the left, *a b*, of a 

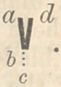

. Then pass the knife again into the vagina, still dilating with the fingers as before, and cut in like manner on the opposite side from above downwards, uniting the two incisions at the raphe, as shown by the line *d b*, which is to be extended quite to the perineal integument, and through its upper border, as shown by the dotted line *b c*. Each cut will be nearly two inches long, extending from about half-an-inch above the upper border of the sphincter vaginæ, across the sphicter for about half-an-inch, and down to the perineal raphe for nearly an inch more. Of course, this will vary in different subjects, according to the development of tissue in each. To perfect the cure, the patient will wear for a time a properly adapted vaginal dilator. I use an instrument usually made of glass, sometimes of silver, or other metal silvered or gilt. I prefer glass, because it is cheap and easily kept clean, while being transparent, it is easy to see how the wound is progressing without removing the instrument. Moreover, some patients have insisted that a glass instrument is more comfortable and less irritating than one of metal. I am not prepared to say whether this be true, yet there may be both truth and philosophy in the assertion, as one substance is the worst conductor of heat, and the other among the best. The dilator is sometimes introduced as soon as the operation is finished, especially if there be much hæmorrhage, which always ceases immediately in consequence of the pressure of the instrument. But most generally I do not order it for 24 hours after the operation, when it is worn two, three, or four hours. Its introduction is attended with a sense of soreness, but with none of the peculiar, agonizing suffering, characteristic of the original disease. The instrument is usually worn for two hours in the morning, and two or three hours in the evening, more or less, according to the tolerance of the patient. I have been often astonished at the rapidity with which the cuts heal, the process being seemingly facilitated by the pressure of the glass dilator, which is to be worn daily for two or three hours, or until the parts being entirely cured, and all sensitiveness removed, the patient may be pronounced competent to fulfil comfortably and pleasantly the duty of a wife.

The dilator is about three inches long, sometimes a little more, slightly conical, open at one end and closed at the other, and of different sizes, varying from an inch to an inch and a half in diameter. At the largest part, near the outer extremity, there is a depression on one side for the urethra and neck of the bladder. It is open at the outer end, to allow the pressure of the atmosphere to hold it in the vagina, which it does very effectively. When closed at both ends, a T bandage is necessary, and the instrument often slips. I found that a perfectly round cylinder, on being worn for three or four hours, always irritated the urethra and neck of the bladder; hence, the urethral depression on one side, which also materially aids its self-retaining power.

This disease is by no means rare. Dr. Emmet and myself saw seventeen cases in twenty-four months. Of these, one had been married thirty years; one, fifteen years; one, thirteen years; one, seven years; one, six years; three, three years; and so on down to two years. Of these, fifteen had been treated, all of whom were cured. Three have become mothers—one conceiving in two months after her cure, one in four months, and another in twenty months—and I have no doubt that many more will become mothers in due course of time. In most of them, sexual intercourse had never been accomplished; in two, it had been done a few times very imperfectly, then suspended altogether; while in two others it had been indulged in under the most trying circumstances, and always with dreadful suffering to the wife, and in these there was the most complete wreck of the nerves, if such an expression may be allowed. All were married but one. In this case, the affection was not discovered until her physician made an effort to find out something about the state of her womb, as she was suffering greatly from dysmenorrhœa. He then sent her to me, supposing that she had atresia vaginæ. The vaginismus was cured to two or three weeks, after which the patient returned to her physician for treatment of her dysmenorrhœa.

It must not be for a moment supposed that I arrogate to myself the discovery or description of a new disease. I do not, for it has been encountered for all time. I claim only to have separated Vaginismus from a great class in which it had been obscurely hidden away. Others have met it before. Some have called it neurosis; but this is a generic term, which may be applied to any painful affection, uncomplicated with inflammation. Many have called it neuralgia; but this term is wholly inapplicable, for it has none of its habitudes. Neuralgia is supposed to be a painful affection usually in the course of a nerve, coming when it pleases, remaining as long as it pleases, and disappearing when it pleases, but usually observing a particular cycle of time in its advent, its culmination, and its decline. Let it once leave and it cannot be recalled at will; but vaginismus can be provoked at any moment by the gentlest touch, ceasing immediately on removing the irritating cause, never returning spontaneously, and never returning at all except under the same mechanical agency. Time will show that this is not the only disease where our ignorance is covered over by the broad mantle of neuralga. Some have called it hyperæsthesia, but this is only another phase of neuralgia—a thing that is here today and gone to-morrow, and is most generally symptomatic of some other affection. I call it Vaginismus, because it is not only a symptom but a conglomeration of symptoms, constituting a distinct and separate disease, with as good a right to a proper name as any disease enumerated in our nosology.

If, by the invariable uniformity of symptoms, if by the frightful amount of physical, moral, and social suffering which it always engenders, or, better still, if, by the certainty, facility, and safety of its treatment, a disease be entitled to a particular name and special study, then must Vaginismus be hereafter recognised whenever seen, and cured whenever treated.—*American Medical Times.*

